# Influence of the Welding Degree on the Strength and Failure Modes of Tuff

**DOI:** 10.3390/ma15248757

**Published:** 2022-12-08

**Authors:** Lihui Li, Chenglong Li, Beixiu Huang, Ming Wang, Zhida Bai, Shengwen Qi

**Affiliations:** 1 Key Laboratory of Shale Gas and Geoengineering, Institute of Geology and Geophysics, Chinese Academy of Sciences, Beijing 100029, China; 2 Innovation Academy for Earth Science, Chinese Academy of Sciences, Beijing 100029, China; 3 College of Earth and Planetary Sciences, University of Chinese Academy of Sciences, Beijing 100049, China; 4 College of Geoscience and Surveying Engineering, China University of Mining and Technology, Beijing 100083, China; 5 Shenzhen Institute of Advanced Technology, Chinese Academy of Sciences, Shenzhen 518055, China; 6 School of Earth Sciences and Resources, China University of Geosciences, Beijing 100083, China

**Keywords:** welding degree, tuff, mechanical property, strength, failure mode, diagenesis

## Abstract

The diagenesis of welded tuffs is a process in which volcanic debris undergoes degassing, compaction, and quenching, and vitreous rheologic, which indicates that the welding occurred in a high-temperature, high-pressure diagenetic environment and that different temperatures and pressures result in different degrees of welding in the welded tuffs, which can also result in differences in the mechanical properties of the rock. In this study, based on petrographic identification, mineral composition analysis, and pore structure characterization, uniaxial compression combined with linear accelerator CT and Brazilian splitting tests was carried out to investigate the influence of the welding degree on the strength and failure modes. The test results showed that although they had almost similar mineral composition and porosity, the uniaxial compression strength and tensile strength of the strongly welded tuffs were greater than that of the weakly welded tuffs. Their failure modes were also different. Fractures in the weakly welded tuffs developed gradually, while the strongly welded tuffs showed a higher brittleness with sudden failure. The results of this study shed light on the influence of the diagenetic environment on the mechanical properties of rock from a geological perspective and can provide a mechanical basis for rockfall risk evaluation in scenic areas of welded tuff.

## 1. Introduction

Welded tuffs are one of the most important types of volcanic clastic rocks, which are mostly formed by the accumulation of volcanic debris flows from intense volcanic eruptions. The main types of volcanic debris in welded tuffs are rigid debris–detritus and crystal fragments, plastic debris–glass shards glass shards, magma fragments, pumice fragments, and cemented volcanic dust [1,2]. The main minerals comprising welded tuff are quartz, feldspar, muscovite, and pyrite, which account for over 90% of the total. Welded tuffs have a welded tuff texture and a pseudo-rhyolitic structure [3,4,5]. Welded tuffs can be defined as strongly welded tuffs, medium welded tuffs, and weakly (i.e., plasticized) welded tuffs, depending on the degree of deformation of the glass shards [6,7]. In the accumulation of volcanic debris flows in a cooling unit, welding occurs mainly in the middle of the cooling unit, with a weak–strong–weak variation in the degree of welding [8,9]. In strongly welded tuffs, there are a large number of glass shards with severe deformation, and a pseudo-rhyolitic structure develops. In weakly welded tuffs, the glass shards are crescent, bow shaped, arched triangular, slightly deformed, and poorly developed with pseudo-rhyolitic structures [10,11].

The diagenesis of welded tuffs is a process in which volcanic debris undergoes degassing, compaction, quenching, and vitreous rheologic [12,13,14]. This indicates that the welding occurred in a high-temperature, high-pressure diagenetic environment, and different temperatures and pressures result in different degrees of welding in welded tuffs, which also leads to differences in the mechanical properties of the rock [15]. Geissman et al. [16] found that tuffs had large-scale rheological structures and plastic deformation in response to high temperatures during diagenesis. The rock structure changes as a result of the differences in the diagenetic environment. Branney and Sparks [17] suggested that pseudo-rhyolitic structures are formed by the directional arrangement of compressed and elongated plastic glass shards and plastic detritus, forming discontinuous structural surfaces as the volcanic debris welds. The mechanical behavior of welded tuffs is more complex than that of conventional homogeneous isotropic materials due to the different welding degrees and discontinuous structures [18].

In a study on Brisbane welded tuff, Erarslan et al. [19] and Erarslan and Williams [20] observed that intergranular cracks and transgranular cracks formed smooth and bright fractures on the cleavage plane of the rock after the failure of the tuff under static loading. Sandeep et al. [21] found that the tuff had very high friction angles at the grain contacts compared to other materials after studying the contact mechanics and tribological behavior of highly decomposed tuff grains. Price and Bauer [22,23] found that variation in the effective porosity significantly affected the unconfined compressive strength and Young’s modulus of the rock and that the Poisson’s ratio was significantly influenced by the effective porosity and particle density in tests on the mechanical properties of rocks from welded tuffs in Nevada, USA.

To explore the effects of different temperature and pressure environments on the mechanical properties of rocks, previous researchers have conducted numerous studies on the microstructural evolution of rocks of different lithologies (e.g., granite, sandstone, and limestone) at different temperatures using acoustic emission monitoring [24,25,26], microscopic thin section observation [27], scanning electron microscope analysis [28,29,30], and CT scanning [26].

The previous research has usually been conducted under strata temperature conditions (20 °C to 150 °C) for pressurization tests to simulate the real strata pressure and temperature environment. The mechanical parameters, such as compressive strength, shear strength, and residual strength, of the rocks increased with the temperature and pressure, but the variations in the elastic modulus and Poisson’s ratio exhibited different trends depending on the lithology [31,32]. Fang and Wu [33] concluded that the mechanical properties of rocks at different temperatures and pressures depended on their original mineralogical composition and structural characteristics. By comparing the mechanical properties of welded tuffs before and after high-temperature treatment, it was observed that the strength of the rocks decreased with the increasing temperature, which became more obvious at higher temperatures [34,35]. Rutqvist and Tsang [36] indicated that the major reason for the variation in the mechanical properties of rocks was the thermal expansion of the rock from 200 °C, and the thermal expansion coefficient depended on the temperature. The explanation for the decrease in the strength of tuffs at high temperatures was that the decomposition of the thermally unstable minerals [37] melts the fracture, and the recrystallization of minerals alters the initial mineralogical composition of the rock, which affects the deformation and failure characteristics of the tuffs as well.

However, tuffs with different welding degrees formed at different temperatures and pressures owing to their location in different flow units during diagenesis, which may influence their structure and strength and then the stability of the high-steep slope. Field investigations on steep tuff outcrops in rockfall-prone areas showed that the tuff had a large range of joint densities [38,39]. In the Shenxianju Scenic Area, some welded tuff outcrops were characterized by low density and very large blocks, while others had a high joint density and very small blocks. This suggests that the different mechanical properties of rock would influence the stability of the rock mass and, further, the rockfall volume, resulting in different disaster risk degrees. Nevertheless, studies on the influence of the welding degree on the mechanical behavior of tuff are limited, and detailed investigations are in urgent demand. In particular, it is significant to have a thorough evaluation of the disaster risk in scenic areas. Studying the mechanical variety of tuffs can help us figure out why, where, and how rockfalls occurs and effectively prevent a rockfall disaster.

Therefore, we carried out experimental studies on the mechanical properties of tuffs with different welding degrees, with the integration of petrographic identification, mineral composition analysis, and pore structure characterization. We attempted to investigate the influence of the rock formation environment on the mechanical properties of rocks, and we discuss the mechanisms of the differences in the mechanical properties based on the perspective of diagenesis. The findings are very helpful for dynamic rockfall risk analysis and disaster prevention in scenic areas.

## 2. Geological Background

**Figure 1:**
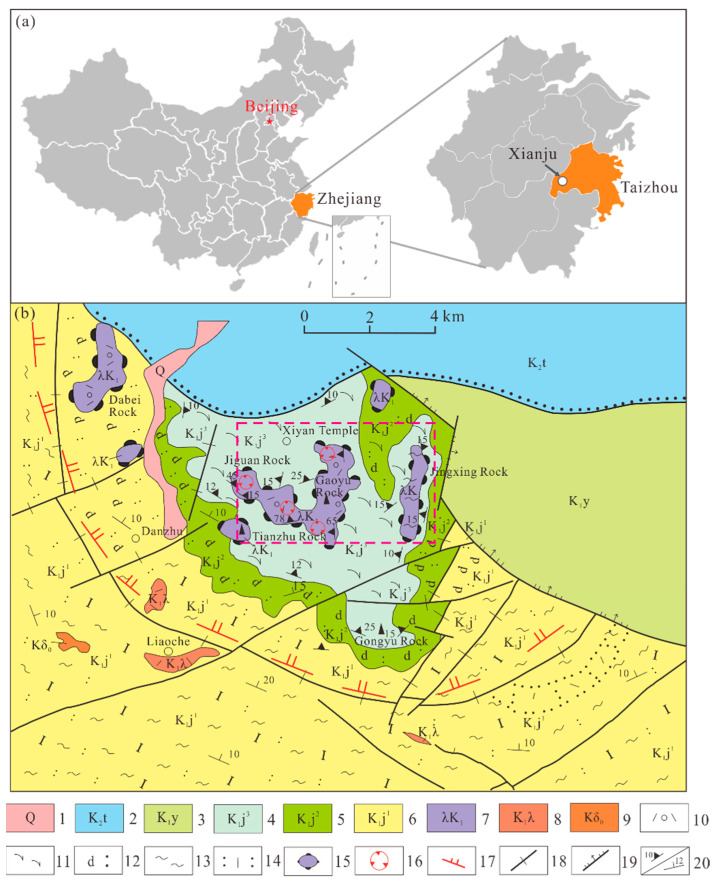
The location and geological map of the volcanic rock facies in the Shenxianju Scenic Area: (**a**) location of Xianju County; (**b**) geological map of the Shenxianju Scenic Area. The carmine-dotted rectangle marks the study area. 1. Quaternary, 2. Upper Cretaceous Tiantai Group; 3. Lower Cretaceous Yongkang Group; 4. third member of the Jiuliping Formation; 5. second member of the Jiuliping Formation; 6. first member of the Jiuliping Formation; 7. intrusive facies rhyolitic foam lava; 8. sub-rhyolite; 9. quartz diorite porphyrite; 10. rhyolitic foam lava; 11. overflow facies rhyolite; 12. Sedimentary tuff intercalated tuffaceous sandstone; 13. Pumice flow facies rhyolitic welded tuff; 15. invading dome; 16. volcanic conduit in the reactivation period; 17. boundary of the caldera; 18. volcanic fault; 19. regional fault; 20. flow surface/strata occurrence.

The samples were obtained from the Shenxianju Scenic Area in Xianju County, Taizhou city, Zhejiang Province (Figure 1a), with a geographical location of 120°33′36.9″~120°40′12.7″ East and 28°35′17.3″~28°43′51.8″ North. The geotectonic position of the Shenxianju Scenic Area was at the active continental margin of the Eurasian plate in the western Pacific Ocean, which belongs to the South China fold system. The Shenxianju Scenic Area is an ancient caldera that was formed in the Early Cretaceous approximately 120 Ma ago, and it is one of the most representative ancient calderas in the volcanic eruption belt along the southeast coast of China.

The caldera shape is incomplete, but the caldera structure is very clear, with an exposed area of approximately 140 km^2^, a diameter of approximately 12 km, and a diameter of a crater approximately 5 km. The stages of volcanic activity are obvious. Large-scale pumice flow accumulation, typical caldera lake volcanic sedimentary rock series, the extremely characteristic resurrection rhyolitic lava, and intrusive rock exposure as well as the development of circular and radial fractures record the complete volcanic eruption process that ended with the strong eruption, collapse, sedimentation, and resurrection to subvolcanic rocks, which, finally, blocked the resurrection channel. It is unique in its spatiotemporal evolution, recording the process of magmatism on the continental margin of the Early Cretaceous, and it an important window for understanding the regional tectonic dynamics of the Early Cretaceous and the deep information of the Earth.

The strata exposed in the study area mainly consist of the Lower Cretaceous Jiuliping Formation (K_1_j), the Lower Cretaceous Yongkang Group (K_1_y), the Upper Cretaceous Tiantai Group (K_2_t), and the quaternary stratum (Q). The major lithology of the Jiuliping Formation is rhyolite and rhyolitic welded tuff with glassy and crystal fragments, locally interspersed with sedimentary tuff and tuffaceous sandstone (Figure 1b).

**Figure 2:**
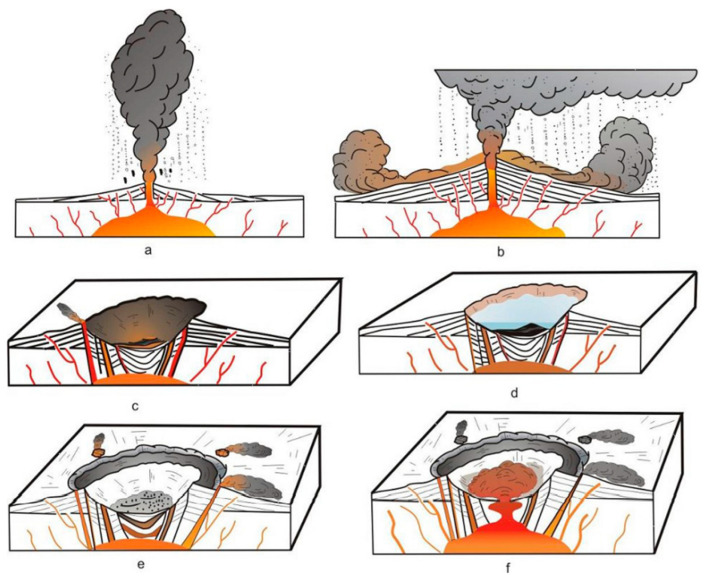
Schematic diagram of the evolution of the Shenxianju caldera: (**a**) initial Britney eruption stage of the caldera, forming the falling accumulation facies; (**b**) large-scale explosion stage, forming the pumice flow due to the collapse of the eruption column; (**c**) magma chamber was evacuated, and the caldera collapsed to form the caldera depression; (**d**) caldera lake formed and began to receive sediments; (**e**) overall collapse of the caldera, the outer ring fracture formed, the caldera revived, and the side crater began to erupt; (**f**) central high-level magma chamber intruded upward, forming the central intrusion. At the same time, an intrusion dome formed above the side crater, blocking the passage and terminating the volcanic activity.

The Shenxianju caldera is an ancient volcano similar to Vesuvius in Italy, the evolution of which is shown in Figure 2. First, a large-scale Britney-style eruption formed tens of kilometers of eruption column above the crater. With the collapse of the eruption column, huge pumice flow accumulations formed, comprising the main body of the volcano. Secondly, after the pumice flow accumulated, due to the volcanic collapse, the caldera formed, the volcanic eruption stopped, and the caldera water created a lake and received deposits, forming a volcanic sedimentary facies rock series. Then, after the volcanic eruption stopped for some time, with the supply and accumulation of the deep magma chamber, the caldera revived again. The magma rose along the circular and radial fractures or the intersection of the fractures, and erupted out of the surface, forming a series of secondary revived volcanoes. Then, due to the insufficient supply of magma, the viscosity increased, and the volcanic activity was nearing its end. Finally, high-viscosity magma extruded or invaded the channel and later blocked the volcanic channel, ending the whole process of volcanic activity. Since the late Early Cretaceous, the volcano has been in a stage of denudation and transformation.

**Figure 3:**
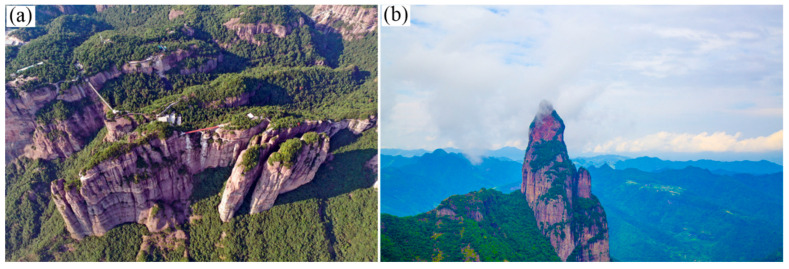
The beautiful landscapes in the Shenxianju Scenic Area: (**a**) cliffs with a height difference of almost 700 m; (**b**) rock spine like the Buddhism goodness Guanyin.

Due to the joint action of the regional fault activity and weathering denudation, the original terrain of the caldera was destroyed, and the Shenxianju caldera only retained a part of the crescent moon shape. With the intensification of the fracture cutting and the interaction of weathering, denudation and river erosion, the current terrain was gradually shaped, forming various beautiful volcanic rock landscapes, as shown in Figure 3.

## 3. Samples and Methods

**Figure 4:**
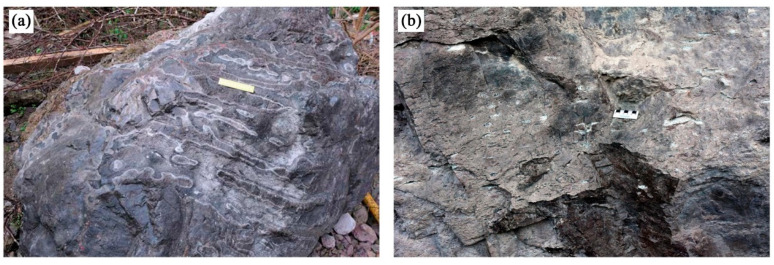
The welded tuffs of the Lower Cretaceous Jiuliping Formation: (**a**) strongly welded tuff; (**b**) weakly welded tuff.

The rock samples were strongly welded tuff and weakly welded tuff collected from the Shenxianju Scenic Area, as shown in Figure 4, which belonged to the Lower Cretaceous Jiuliping Formation. The welded tuff exhibited a tuff structure and pseudo-rhyolitic structure, and the mineral orientation could be observed. The color of the fresh rock was flesh-red, with a few variations in the color depending on the degree of welding. The weakly welded tuffs consisted of a large amount of clastic material, such as glass shards and crystal fragments; the clasts were directional, and there were clear boundaries between them. The clasts were weakly cemented to each other. The strongly welded tuffs had long strips and ribbon-shaped dark magma fragments interspersed with crystal fragments, glassy shards closely cemented with volcanic ash and indistinguishable because of the welding. The oriented glass shards were curved by lithic clasts and crystal fragments, resulting in a pseudo-rhyolitic structure. The strongly welded tuffs had a clearer pseudo-rhyolitic structure than the weakly welded tuffs.

### 3.1. Petrographic and Mineralogical Identification

The petrographic features of the tuff were achieved by thin section identification. The samples of the welded tuff were cut into thin slices that were 30 μm thick and then polished. These slices were analyzed using a polarized light microscope Leica DM4P to identify the mineralogical and textural features. Meanwhile, the samples were powdered, passed through a 200-mesh sieve, and analyzed by a multifunctional Rigaku TTRIII X-ray diffractometer to characterize their mineralogical compositions. The weight percentage of the minerals was measured according to the standard analysis method (SY/T 5163-2018) for clay minerals and ordinary non-clay minerals in sedimentary rocks by X-ray diffraction [40].

### 3.2. Pore Measurements and Calculations

The pore structure of the welded tuffs was determined by a combination of nuclear magnetic resonance (NMR) and mercury intrusion porosimetry (MIP) tests. A Magritek 2 MHz nuclear magnetic resonance (NMR) core analyzer was applied to measure the pore size distribution of the welded tuff samples (cylinders 25 mm in diameter and 50 mm in height). The instrument applied a CPMG (Carr–Purcell–Meiboom–Gill) radio frequency sequence, setting 4000 echoes in a CPMG echo train and a 60 μs echo spacing. The collected data were finally subjected to Gaussian inversion to obtain the T_2_ curves of the welded tuff specimens. The MIP test was conducted on cylindrical specimens 20 mm in length and 10 mm in diameter. The mercury in the nonwetting phase entered the smaller pores under the action of external forces. The Washburn equation, Equation (1), was used to calculate the pore radius [41]. More details can be found in Li et al. [42].

(1) $$r=\frac{2\sigma cos\alpha }{p_{c}}\;\;\;\;\;\;\;\;\;\;\;\;\;\;\;\;\;\;\;\;\;\;\;\;$$

where *r* is the pore radius, *p_c_* is the mercury injection capillary pressure, *α* is the mercury contact angle, and the *σ* is the surface tension.

### 3.3. Mechanical Tests

**Figure 5:**
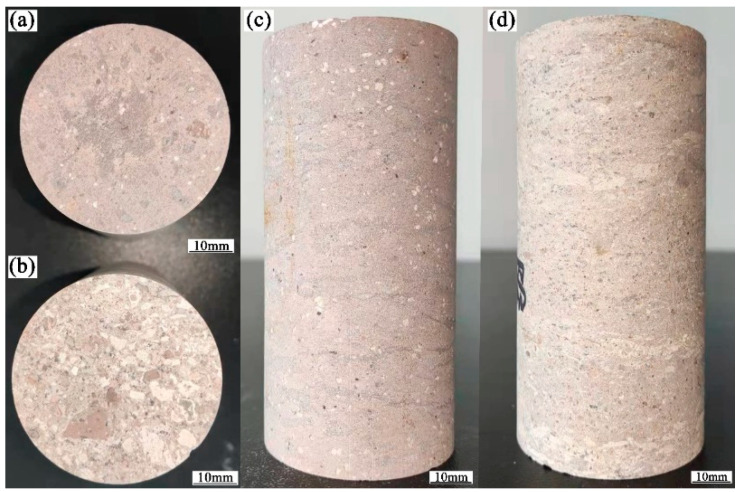
The tuff samples with various welding degrees were prepared for the mechanical tests: (**a**,**b**) the discs of strongly and weakly welded tuff for the Brazilian splitting tests, respectively; (**c**,**d**) the standard cylindrical specimens of strongly and weakly welded tuff for uniaxial compression tests, respectively.

The rock mechanics experiments in this study included uniaxial compression tests and Brazilian splitting tests. The samples were prepared according to the International Society of Rock Mechanics (ISRM) recommended standards [43]. The specimens were standard cylindrical specimens of 50 mm in diameter and 100 mm in height for the uniaxial compression and standard disc specimens of 50 mm in diameter and 25 mm in thickness for Brazilian splitting tests (Figure 5).

To avoid the possible influence of the pseudo-rhyolitic structure on the mechanical properties of the rock, the loading angle of the prepared samples was uniformly taken as β = 90°. The size deviation of an ideal specimen was ±0.1 mm, with a parallel misalignment between the top and bottom faces of ±0.02 mm and an axial angle deviation of less than 0.05°. In the uniaxial compression tests, five samples of each type were tested in the conventional procedure. The loading method for the cylinders in the test was a constant loading rate of 0.8 kN/s. The discs were loaded at a constant displacement rate of 0.35 mm/min until failure in the Brazilian splitting tests. In this study, the samples after uniaxial compression experiments were scanned using the geomechanics test system with a linear accelerator CT, and fracture information was obtained using VGStudio MAX 2.1 software.

## 4. Mineralogical, Petrological, and Porosity Characteristics of the Samples

### 4.1. Petrographic Identification Characteristics

**Figure 6:**
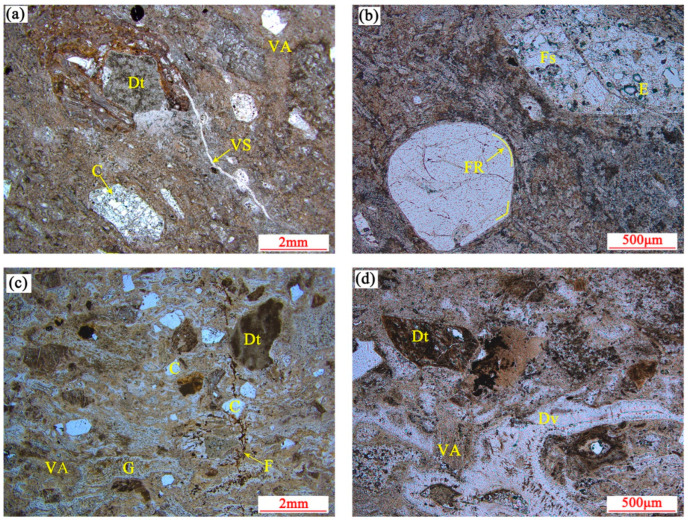
Plane-polarized photomicrographs of welded tuffs: (**a**,**b**) strongly welded tuff; (**c**,**d**) weakly welded tuff. C: crystal fragment; Dt: detritus; Dv: devitrification; E: erosion pores; F: fissure; FR: fusion rounding; Fs: feldspar; G: glass shards; VA: volcanic ash; VS: veined silica strips.

The strongly welded tuffs were formed in the depth of the stratum under pressure from the overlying strata, with temperatures of 650 to 700 °C as diagenesis. From the thin section, it can be seen that the strongly welded tuff was mainly composed of plastic glass shards and magma debris fragments mixed with a small amount of rigid debris (Figure 6a). The magma fragments were strongly flattened and elongated and arranged in parallel, often in the form of bands or long lenses. The plastic glass shards were generally elongated into ribbons, forming a pseudo-rhyolitic structure together with the magma fragments. Some glass shards had been devitrified to form felsic minerals with local shear deformation. The rocks contained erosion pores that were mostly found inside the feldspar crystals (Figure 6b). The crystal fragments were rounded because of the high-temperature effect. The samples contained microfractures that were filled with silica to form veined silica strips. Severe devitrification of the glass shards occurred. The formation of high-valent iron oxide after the devitrification of the glass shards made the rock present a darker and reddish color.

The weakly welded tuffs were exposed to relatively lower temperatures (500~550 °C) and lower pressures during diagenesis than strongly welded tuffs, with weakly welded structures. The glass shards within the weakly welded tuff were slightly deformed, slightly compressed, and elongated. The basic morphology was still retained with concave angular or chicken bone shapes. The rigid crystal fragments and detritus were angular–subangular with high content. Most of the crystal fragments were quartz. The microfractures were visible within the sample, disrupting the structure of the rigid crystal fragments and detritus (Figure 6c). The devitrification occurred on large volumes of glass shards that exhibited felsic texture inside and a comb-like structure around the edges. The small volume of glass shards was not significantly altered. The mild devitrification occurred on the glass shards of the weakly welded tuffs. The glass shards were in the state of a plastic to rigid transition because the temperature of diagenesis was relatively lower, presenting as semi-rigid (Figure 6d).

### 4.2. Mineralogical Compositions

**Figure 7:**
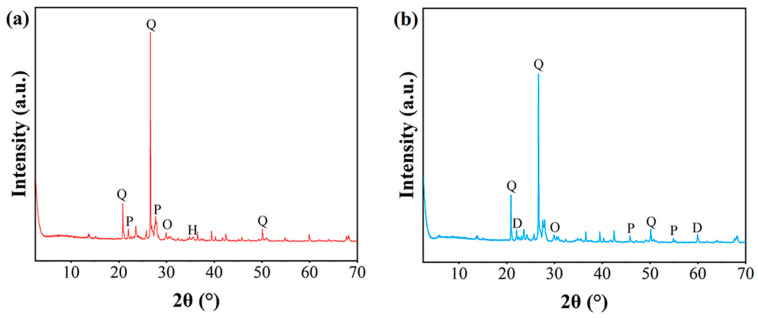
The X-ray diffractograms of the minerals of the welded tuffs: (**a**) strongly welded tuff; (**b**) weakly welded tuff. Q: quartz; O: orthoclase; P: plagioclase; D: dolomite; H: hematite.

X-ray diffraction was conducted on the welded tuff to analyze the mineralogical composition, as shown in Table 1. The X-ray diffractograms of the minerals are shown in Figure 7. Quartz with a content of approximately 70% was dominant, followed by orthoclase and plagioclase with a content of approximately 25%. Dolomite, hematite, and clay minerals were present as subordinate minerals. Dolomite, hematite, and clay minerals were subordinate minerals. The weakly welded tuff and the strongly welded tuff exhibited a high consistency in the content of the quartz and feldspar, which was probably due to the source of the debris material, especially the magma fragments ejected during the eruption of the caldera, and the debris of the surrounding rocks inside the volcanic conduit was similar.

There was a clear difference in the content of the minor minerals, such as dolomite, hematite, and clay minerals, which probably resulted from the instability of the dolomite in a high-temperature and -pressure environment, and the high-valent iron released by the devitrification of the glass shards formed hematite. Smectite minerals dominated in the weakly welded tuffs, and clay minerals in the strongly welded tuffs were dominated by an illite/smectite mixed layer, which resulted from the smectite illitization mostly commencing at 100~130 °C.

### 4.3. Pore Structures

**Figure 8:**
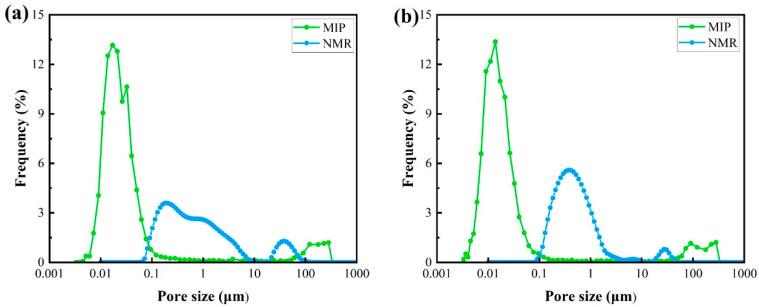
The pore size distribution was measured using nuclear magnetic resonance (NMR) and mercury intrusion porosimetry (MIP) of the welded tuffs: (**a**) strongly welded tuff; (**b**) weakly welded tuff.

In our study, a combination of nuclear magnetic resonance (NMR) and mercury intrusion porosimetry (MIP) data provided information regarding the pore structure. The T_2_-mapping of the NMR reflected the pore structure, and the peak area of the NMR signal indicated the pore volume. The MIP method was based on a cylindrical pore model. Mercury at room temperature was pressed into the capillary pores at the given pressure, and the volume of mercury entering the pore was the volume of the pore to which the throat was connected. Therefore, the distribution of spherical pores in the pore structure was determined by the NMR technique, and the distribution of cylindrical pore throats in the pore structure was determined by the MIP technique, as shown in Figure 8.

The porosity and average pore diameter of the tuffs were calculated, with a porosity of 8.25% and average pore diameter of 22.49 nm for the strongly welded tuff and 8.41% and 14.98 nm for the weakly welded tuff, respectively. The pore size distribution statistics were performed using the classification criteria suggested by Keller and Staudt [44]. This criterion categorized the pores by aperture as ultra-micropores (<0.6 nm), micropores (0.6–2 nm), mesopores (2–50 nm), macropores (50 nm to 2 μm), capillaries (2–50 μm), and micro capillaries (>50 μm). The spherical pores in the strongly welded tuffs were distributed in macropores and capillaries, and the spherical pores in the weakly welded tuffs were dominated by macropores. The pore throat diameter in the welded tuffs was an order of magnitude smaller than that of the pore body itself and was dominated by mesopores. There was speculation that the capillary pores appear in the strongly welded tuffs because of the appearance of the erosion pores in the rock in high-temperature and high-pressure environments during diagenesis (Figure 6b). It was also not excluded that the reason for the devitrification of the glass shards was to produce devitrification pores. The differences in the pore size distribution between the two tuffs were not great, and there was almost no difference in the total porosity.

## 5. Mechanical Properties of the Welded Tuffs with Different Welding Degrees

### 5.1. Strength of the Welded Tuffs

**Figure 9:**
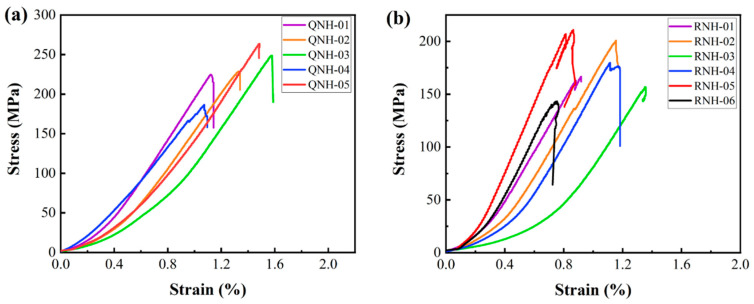
Stress–strain curves of a uniaxial compression test: (**a**) strongly welded tuffs; (**b**) weakly welded tuffs.

The stress–strain curves of the welded tuff were obtained by conventional uniaxial compression tests, as shown in Figure 9. There was a longer initial compaction stage in the curve of the strongly welded tuff, and it was speculated that the closure of the erosion pores or devitrification pores occurred in the rock at the initial compaction stage. Some of the weakly welded tuffs also exhibited an obvious compaction stage, which was presumed to be the result of the closure of microfractures in the rock, as shown in the thin section identification (Figure 6c). With the increased loading, the elastic stage began and was then followed by thorough fracture, i.e., the brittle failure occurred ultimately.

The mechanical test results of the welded tuffs are shown in Table 2. The peak strength of the strongly welded tuffs ranged from 186.76 MPa to 263.75 MPa, with an average of 230.57 MPa, and the elastic modulus ranged from 1.16 GPa to 1.64 GPa, with an average of 1.41 GPa. The peak strength of the weakly welded tuffs ranged from 143.33 MPa to 210.92 MPa, with an average of 176.42 MPa, and the elastic modulus ranged from 0.79 GPa to 2.13 GPa, with an average of 1.43 GPa. The tensile strength of the strongly welded tuffs ranged from 10.82 MPa to 12.14 MPa, with an average of 11.64 MPa. The tensile strength of the weakly welded tuffs ranged from 8.35 to 14.33 MPa, with an average of 10.98 MPa.

The results demonstrated that both the uniaxial comprehensive and tensile strength of the strongly welded tuff were higher than the rock strength of the weakly welded tuff. The differences in the elastic modulus between the two kinds of tuffs were slight. However, the results of the mechanical parameters of the weakly welded tuff specimens were strongly discrete, indicating that the weakly welded tuffs were more heterogeneous than the strongly welded tuffs, which might have been induced by rigid rock debris in the weakly welded tuff. From the stress–strain curve, the post-peak curve of the weakly welded tuff showed fluctuations, suggesting that rigid rock debris formed the rock bridges and prevented the development of transgranular fractures.

### 5.2. Failure Modes of the Welded Tuffs

**Figure 10:**
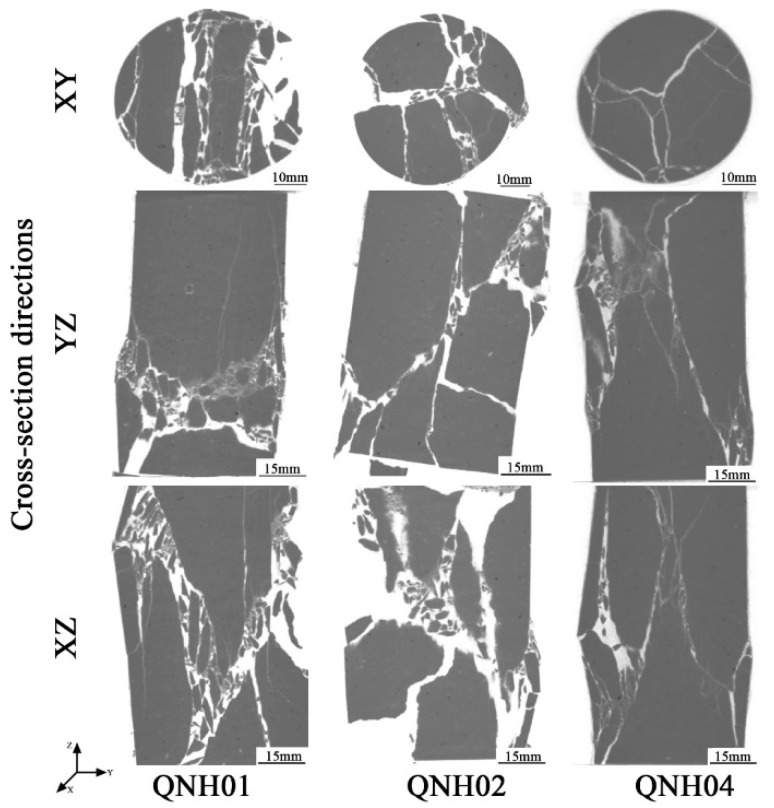
Three corresponding views of the strongly welded tuff specimens were scanned with a linear accelerator CT. From top to bottom, the cross-sections are of the X-Y plane (parallel to the bottom of the sample), the Y-Z plane, and the X-Z plane, respectively.

**Figure 11:**
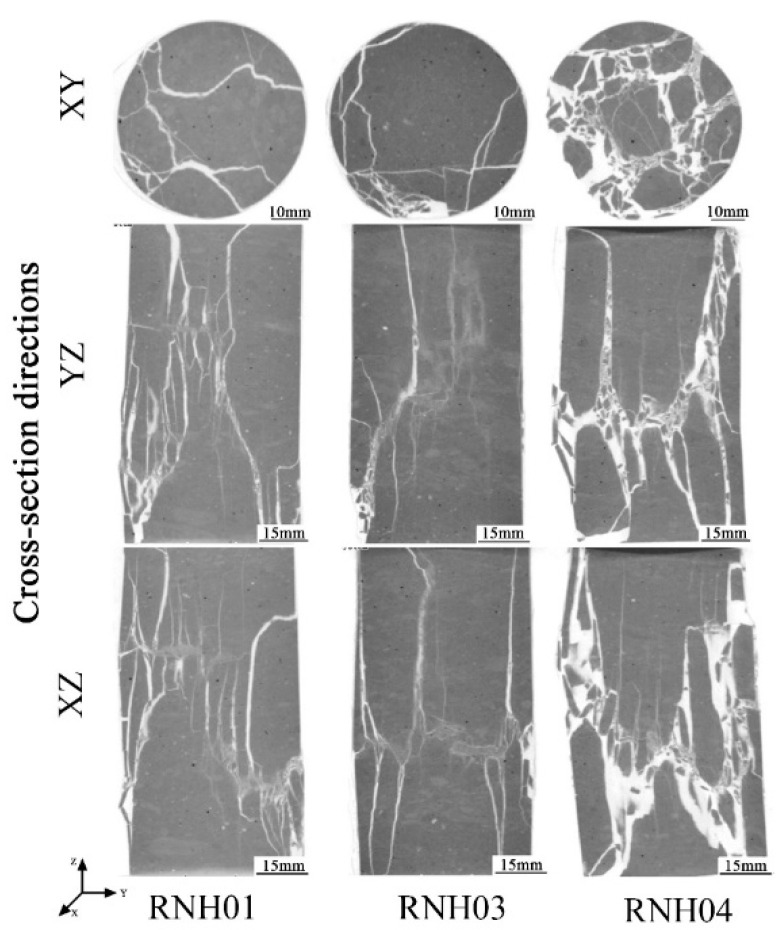
Three corresponding views of the weakly welded tuff specimens were scanned with a linear accelerator CT. From top to bottom, the cross-sections are of the X-Y plane (parallel to the bottom of the sample), the Y-Z plane, and the X-Z plane, respectively.

The samples of welded tuffs after the uniaxial compression tests were scanned by linear accelerator CT technology. The successive slices obtained from the scans were imported into VGStudio Max V2.1 software to create a volume object with a spatial resolution of 0.129 mm/voxel. Then, the surface determination was performed to differentiate the background and material according to the gray value. After, the region of interest (ROI) was created, and the borders of the ROI were adjusted using gray value gradients. The internal fracture was extracted by conducting a porosity/inclusion analysis and filtering (Figure 10 and Figure 11).

The internal fractures of the strongly welded tuffs are presented in Figure 10. Two through shear fractures appeared in the crushed sample of QNH01, with a dip angle of approximately 60°. The presence of a multitude of tiny, fragmented clasts inside the fractures implied that continuous longitudinal tensional fractures occurred before the development of the shear fractures. The fractures of the QNH02 sample exhibited different orientations, but the dip angle of the main fracture still exhibited approximately 60°. The fracture extension of the sample was perhaps mainly controlled by the microfractures. The failure of the QNH04 specimen was a conical rupture. Under the control of tensile deformation, it consisted of some small longitudinal fractures converging into two major fractures.

Figure 11 shows the CT scanning results of the weakly welded tuffs. From the figure, it was inferred that the failure process probably started with the appearance of small axial tensile fractures inside the specimen of RNH01, and then a series of small tensile fractures suddenly converged into a shear band under the ultimate load.

The failure type of the RNH03 sample was the same as that of RNH01, but there were additional tensile fractures parallel to the axial direction of the specimen and through the sample, which was a kind of tensile–shear failure mechanism. There should be some microcracks in the sample that affected the development of transverse cracks. This was consistent with the longer compaction stages observed in the stress–strain curve.

The RNH04 sample had more fractures and broke into more fragments, but the approximate direction of the main fracture could be observed, presenting an angle of dip of approximately 60°. The main fracture was composed of small longitudinal tensile fractures. The sample accumulated energy during the loading process and then suddenly underwent brittle failure after reaching the peak strength.

**Figure 12:**
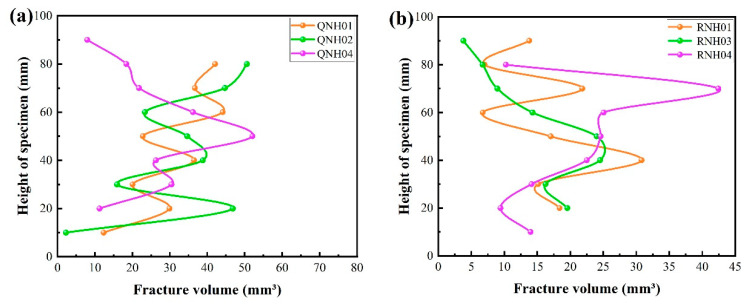
Distribution of the fracture volume along with the height of the specimen: (**a**) strongly welded tuffs; (**b**) weakly welded tuffs.

For further study, the spatial distribution of the fracture volume (i.e., the fracture volume along the height of the specimen) was achieved as shown in Figure 12. For the strongly welded tuff, the volume of fractures at different heights was relatively uniform. This phenomenon corresponded to the failure mode of the strongly welded tuff, as illustrated in Figure 10, which failed with main fractures that penetrated the top and bottom. It also suggests that the samples presented sudden brittle damage at peak strength, as demonstrated by the stress–strain curve shown in Figure 9a. In addition, the main fractures penetrating the top and bottom resulted in a larger fracture volume relative to the weakly welded tuffs. Although the fracture volume of the weakly welded tuffs was relatively lower, the differences in the spatial distribution of the fractures within parallel samples were more pronounced. The weakly welded tuffs had many tiny fractures that were generated from weak structural planes within the rock and failed with multiple-stage damages. This was consistent with the appearance of small steep bumps before peak strength in the specimens of weakly welded tuffs (Figure 9b), and the CT images in Figure 11 also illustrate the morphology of the sample after failure by microcrack extensions.

## 6. Discussion

The results of the uniaxial compression test and Brazilian splitting test on the welded tuffs showed that the strength of the strongly welded tuffs was greater than that of the weakly welded tuffs in general. The CT scanning tests demonstrated the failure difference between the strongly welded tuff and the weakly welded tuff under uniaxial compression tests. The strongly welded tuffs tended to develop more obvious primary fractures, with the higher energy accumulated during compression being released at rupture. The weakly welded tuffs developed more microfractures internally after failure. The failure mode of the welded tuffs was mostly shear failure formed by the convergence of multiple tiny tensor fractures.

From the mineral composition and porosity of the two kinds of welded tuff, the weakly welded tuff and the strongly welded tuff exhibited a high consistency in the content of the quartz and feldspar. The differences in the pore size distribution between them were not great, and there were almost no differences in the total porosity. However, the thin section identification showed that their microstructure differed significantly due to the different temperature and pressure environments during diagenesis. Their mechanical properties (i.e., strengths) were different and with different failure modes. The fracture volume of the strongly welded tuff was larger than that of the weakly welded tuff, and small fractures expanded into main fractures at the same time. Fractures in the weakly welded tuff gradually developed.

It could be inferred that the strength of the welded tuff was mainly controlled by the cementation, while the failure modes were mainly influenced by the content and distribution of the glass shards during the diagenetic process. The glass shards within the weakly welded tuffs were mostly semi-rigid. The distribution of the volcanic debris inside the weakly welded tuffs was uneven and the cementation force was weak. The glass shards within the strongly welded tuffs were plastic. The rock was relatively well homogeneous and tightly cemented with volcanic debris. The rigid debris also influenced the mechanical properties of the rock, which was reflected in the higher strength required for the fracture expansion into the rigid debris interior during rupture. In summary, the temperature and pressure of the diagenesis determined the degree of welding of the welded tuffs, the mechanical properties of the glass shards, and the degree of cementation between the volcanic debris. This may be the reason for the significant variability in strength and failure modes.

Combining the strength, failure mode, and fracture volume of the tuff with different welding degrees, it is supposed that the rockfall blocks of the strongly welded tuff may have a larger volume and lower density, while in the weakly welded tuff, they may be characterized by a smaller volume and higher density. In other words, the welded tuffs of the different welding degrees had different mechanical properties and different stabilities of the rock mass, which results in various rockfall risk degrees and demands different prevention measures.

## 7. Conclusions

The weakly welded tuff and the strongly welded tuff exhibited a high consistency in the content of the quartz and feldspar. The differences in the pore size distribution between them were not great, and there was almost no difference in the total porosity.

The uniaxial compression strength and tensile strength of the strongly welded tuffs were greater than that of the weakly welded tuffs. Their failure modes were also different. The fractures in the weakly welded tuff developed gradually, while the strongly welded tuff showed higher brittleness with sudden failure.

The higher the degree of the welding, the higher the temperature and pressure at the time of the diagenesis. The temperature and pressure at the time of the diagenesis determined the degree of welding of the welded tuffs, the mechanical properties of the glass shards, and the degree of cementation between the volcanic debris. This may be the reason for the significant variability in the strength and failure modes.

## Figures and Tables

**Table 1:** The mineralogical composition of the welded tuff.

Lithology	Mineral Contents (%)											
	Q	O	P	D	H	CM	Relative Content of Clay Minerals (%)					
							S	I/S	I	K	C	C/S
Strongly welded tuff	68.5	8.2	18.7	/	1.7	2.9	/	87	13	/	/	/
Weakly welded tuff	69.1	11.5	12.8	2.8	/	3.8	88	/	12	/	/	/

Q: quartz; O: orthoclase; P: plagioclase; D: dolomite; H: hematite; CM: clay mineral; S: smectite; I/S: illite/smectite mixed layer; I: illite; K: kaolinite; C: chlorite; C/S: chlorite/smectite mixed layer.

**Table 2:** The results of the mechanical properties of the different welded tuff.

Sample	No.	Uniaxial Compressive Strength (MPa)	Average	Elastic Modulus (GPa)	Average	Tensile Strength (MPa)	Average
Strongly welded tuff	QNH01	224.89	230.57	1.64	1.41	12.00	11.64
	QNH02	228.75		1.37		11.72	
	QNH03	248.68		1.16		11.53	
	QNH04	186.76		1.52		10.82	
	QNH05	263.75		1.38		12.14	
Weakly welded tuff	RNH01	166.81	176.42	1.52	1.43	10.99	10.98
	RNH02	200.95		1.39		14.33	
	RNH03	156.78		0.79		9.46	
	RNH04	179.74		1.2		11.79	
	RNH05	210.92		2.13		8.35	
	RNH06	143.33		1.54		/	
